# A novel culprit in glucose hypometabolism in Alzheimer’s disease

**DOI:** 10.1002/alz.095074

**Published:** 2025-01-09

**Authors:** Ylauna Penalva, Marina Ruelas Hernandez, Jessica Cinkornpumin, Mari Kaartinen, William Pastor, Lisa Munter

**Affiliations:** ^1^ McGill University, Montréal, QC Canada; ^2^ McGill Univeristy, Montréal, QC Canada

## Abstract

**Background:**

Alzheimer’s disease is characterized by early decreases in cerebral glucose metabolism which are linked to reduced glucose transporter 1 (GLUT1) expression at the blood‐brain barrier (BBB). Another key disease hallmark is the abundance of Aβ peptides as plaques in the brain which arise from the processing of the amyloid precursor protein (APP). Autosomal dominant inherited mutations causatively link APP itself to AD, rendering it imperative to fully understand APP’s physiological functions to define the underlying biology of AD. Previous studies in APP knockout (KO) mice implicate APP in metabolic processes such as mitochondrial function and insulin action. However, GLUT1 regulation as well as glucose metabolism at the BBB has yet to be investigated.

**Method:**

We use APP KO mouse embryonic fibroblasts (MEFs), mouse primary cerebral endothelial cells, and vessels enriched from mouse brains to study GLUT1 physiology. RNAseq data was collected in MEFs to determine transcriptional changes underlying studied phenotypes.

**Result:**

Seahorse analysis of APP KO MEFs revealed a 50% decrease in glycolytic activity and 75% decrease in mitochondrial respiration as compared to control. Glucose uptake was also significantly decreased in the absence of APP. These metabolic defects were underlined by a 2‐fold decrease in GLUT1 total expression and a 5‐fold decrease in GLUT1 cell surface levels. The investigation of signaling pathways responsible for GLUT1 upregulation showed that PI3K/Akt/mTORC pathway was downregulated in APP KO MEFs as compared to control. Primary cerebral endothelial cells and vessels recapitulated the decrease in GLUT1 expression as well as decreased activation of the PI3K/Akt/mTORC pathway, suggesting a completely novel role for APP in cerebral glucose metabolism. Analysis of RNAseq data collected in MEFs for pathways regulating glucose metabolism revealed widespread changes in extracellular matrix gene expression.

**Conclusion:**

Our findings posit APP as a major regulator of GLUT1 expression and glucose metabolism. This new perspective on APP is crucial for AD therapy as novel approaches could be developed to target decreased cerebral glucose metabolism.

